# Comparative transcriptome analysis of tomato (*Solanum lycopersicum*) in response to exogenous abscisic acid

**DOI:** 10.1186/1471-2164-14-841

**Published:** 2013-12-01

**Authors:** Yan Wang, Xiang Tao, Xiao-Mei Tang, Liang Xiao, Jiao-long Sun, Xue-Feng Yan, Dan Li, Hong-Yuan Deng, Xin-Rong Ma

**Affiliations:** Chengdu Institute of Biology, Chinese Academy of Sciences, No 9, Section 4, Renmin South Road, Chengdu, 610041 China

**Keywords:** Tomato, Exogenous ABA, RNA-Seq, ABA signaling pathway, Transcription factors, Heat shock proteins, Pathogen-related proteins, ROS scavenging enzymes

## Abstract

**Background:**

Abscisic acid (ABA) can regulate the expressions of many stress-responsive genes in plants. However, in defense responses to pathogens, mounting evidence suggests that ABA plays variable roles. Little information exists about genome-wide gene expression in ABA responses in tomato (*Solanum lycopersicum* L.), a model fruit crop plant.

**Results:**

Global transcriptome profiles of tomato leaf responses to exogenous ABA were generated using Illumina RNA-sequencing. More than 173 million base pair reads were mapped onto the tomato reference genome and the expression pattern differences between treated and control leaves were assessed. In total, 50,616 transcripts were generated. Among them, 42,583 were functionally annotated in the NCBI non-redundant database and 47,877 in the tomato genome reference. Additionally, 31,107 transcripts were categorized into 57 functional groups based on Gene Ontology terms, and 14,371 were assigned to 310 Kyoto Encyclopedia of Genes and Genomes pathways. In both the ABA treatment and control samples, 39,671 transcripts were available to analyze their expressions, of which 21,712 (54.73%) responded to exogenous ABA. Of these transcripts, 2,787 were significantly differently expressed genes (DEGs). Many known and novel ABA-induced and -repressed genes were found. Exogenous ABA can influence the ABA signaling pathway with PYR/PYL/RCARs-PP2Cs-SnRK2s as the center. Eighteen PYL genes were detected. A large number of genes related to various transcription factors, heat shock proteins, pathogen resistance, and the salicylic acid, jasmonic acid, and ethylene signaling pathways were up-regulated by exogenous ABA.

**Conclusions:**

The results indicated that ABA has the potential to improve pathogen-resistance and abiotic stress tolerance in tomato. This study presents the global expression analysis of ABA-regulated transcripts in tomato and provides a robust database for investigating the functions of genes induced by ABA.

**Electronic supplementary material:**

The online version of this article (doi:10.1186/1471-2164-14-841) contains supplementary material, which is available to authorized users.

## Background

Tomato (*Solanum lycopersicum* L.) is an important fruit crop and a model system in plants. The basic chromosome number of tomato is 2n = 24, and wild forms range from diploids to hexaploids [1, 2]. The genome of the tomato was recently published; it possesses about 35,000 genes, a rich resource to be studied by scientists [3].

The hormone abscisic acid (ABA) regulates numerous developmental and functional processes, including stomatal aperture and hydraulic conductivity, seed dormancy, a key phase transitions throughout the plant lifecycle [4–6]. An important role of ABA in a variety of plants is to increase tolerance to stresses such as drought, salinity, cold, and heat [7–9]. ABA was recognized as an important signaling molecule that can trigger signaling and regulating mechanisms to cope with adverse stresses. Under drought conditions, plants synthesize and redistribute ABA, leading to guard cell responses that close stomata, thereby reducing plant water loss [10]. Research in chickpea (*Cicer arietinum*) has shown that ABA can improve cold tolerance by, in part, improving the water status of leaves and anti-oxidative ability and can enhance heat tolerance via the accumulation of osmoprotectants [11, 12]. Rice (*Oryza sativa*) seeds pretreated with ABA had improved salt-tolerance, with lower Na^+^ and Cl^–^ levels and Na^+^/K^+^ ratio as well as higher K^+^ and Ca^2+^ concentrations, proline accumulation, soluble sugar content, and grain yield [13].

Mounting evidence suggests that ABA plays contrasting roles during defense responses to pathogens [14, 15]. It interfered at multiple levels with pathogen stress signaling and suppressed or promoted phenylalanine ammonia lyase (PAL) activity [16, 17]. The different plant species assayed in these studies can account, in part, for the various observed effects of ABA on pathogen resistance. ABA is associated predominantly with pathogen susceptibility in tomato, but both negative and positive effects were reported in *Arabidopsis*[18]. Moreover, the synergistic or antagonistic roll of ABA in disease resistance is universally acknowledged to depend on the type of pathogen, its specific way of entering the host, the timing of the defense response, and the type of tissue affected, as well as the concentration of ABA [19]. ABA responses during biotic and abiotic stresses primarily affect levels of gene expression [7]. ABA-induced genes mainly encoded proteins associated with dehydrins and enzymes that detoxify reactive oxygen species, enzymes of compatible solute metabolism, a variety of transporters, regulatory proteins such as transcription factors (TFs), protein kinases and phosphatases, and enzymes involved in phospholipid signaling. ABA-repressed genes usually encode proteins that are associated with growth, including cell wall, ribosomal, plasma membrane, and chloroplast proteins [8].

To comprehensively understand the functions of ABA and related genes, high-throughput screening techniques have proven quite effective [20–23]. Matsui et al. (2008) completed an *Arabidopsis* transcriptome analysis after 2 and 10 h of drought, cold, high-salinity, and ABA-treatment conditions using a tiling array, and the results indicated that approximately 16% of the genes in the transcriptomic library were significantly regulated by ABA. In addition, there was greater crosstalk between ABA and drought and high-salinity stress signaling processes than between ABA- and cold-stress signaling. Similar results were discovered in expression profiles of rice genes under various stresses and ABA application using a cDNA microarray and RNA gel-blot analyses [20]. The impact of ABA on gene expression far exceeds that of other plant hormones. About 3,000 genes detected by microarray were significantly regulated by ABA in *Arabidopsis* seedlings; this number was twice and 23 times as many genes as were influenced by methyl jasmonate and gibberellin, respectively [24].

With the rapid development of next-generation sequencing technology, RNA deep-sequencing (RNA-seq) becomes more efficient and less expensive [25]. The results of RNA-seq are highly reproducible, both technically and biologically [26, 27]. In recent years, numerous transcriptome data sets have been produced, made publicly available, and reanalyzed by other researchers. However, so far, few RNA-seq analyses of the genome-wide responses of genes to ABA in plants have been reported.

We investigated ABA responses in tomato, and here report the results of a comparative transcriptome analysis of exogenous ABA-treated tomato leaves using RNA-seq technology. The goals were to (i) construct a tomato leaf transcriptome; (ii) compare and analyze the transcripts in control and ABA-treated plants; and (iii) gain insight into stress tolerance and pathogen-resistance induced by ABA in tomato. This study presents the transcriptome of tomato leaves responding to ABA and provides a genetic resource that can be used for crop improvement.

## Results and discussion

### Global transcriptome analysis

Tomato seedling sprayed with 7.58 μmol L^-1^ ABA solution were chosen to study ABA responses. Twenty-four hours later, the young third leaves of randomly-selected plants from both the ABA-treated and control groups were collected, labeled as a1d (1 d after ABA-treatment) and c1d (1 d, control), respectively. The tomato ABA/control RNA samples were used for deep sequencing on an Illumina HiSeq 2000 platform. Each read in the Solexa paired-end (PE) sequencing was 101 bp in length. Sequencing generated 266.98 million reads, a total raw dataset of 26.96 Gb. After trimming, 212.78 million clean reads remained, corresponding to 20.95 Gb clean data. The dataset of each sample, including c1d and a1d, was represented by over 100 million reads, a read density sufficient for the subsequent quantitative analysis of genes. The sequence reads were aligned to the tomato reference genome using SOAPaligner/soap2 software (http://soap.genomics.org.cn), allowing two base mismatches [28]. Of the total reads, 81.97% matched either to a unique (36.53%) or to multiple (45.44%) genomic locations (Table 1).

**Table 1:** **Number of reads sequenced and mapped to the tomato genome**

	c1d (control)	a1d (ABA treatment)	Sum
Raw bases (bp)	12,814,237,438	14,141,031,018	26,955,268,456
Raw reads	126,873,638	140,109,218	266,982,856
Clean bases (bp)	9,936,610,838	11,016,491,741	20,953,102,579
Clear reads	100455991	111323699	211779690
Total alignment ^*^ (percent of clean reads)	82394883 82.02%	91194594 81.92%	173589477 81.97%
Unique matches (percent of clean reads)	36742770 36.58%	40622954 36.49%	77365724 36.53%
Multi-position match (percent of clean reads)	45652113 45.44%	50571640 45.43%	96223753 45.44%
Unmatched (percent of clean reads)	18061108 17.98%	20129105 18.08%	38190213 18.03%

*The number of unique mapping reads plus multimapping reads equals the total number of total alignments.

After aligning and assembling, 27,597 genes and 35,051 transcripts were identified in the transcriptome. Of those spliced transcripts, 9,138 (26.07%) matched completely with the annotated tomato genome, 12,501 (35.67%) were potentially novel isoforms, 3,339 (9.53%) were unknown or intergenic transcripts, and 1,634 (4.66%) mapped to the complementary strand of an annotated gene. These results suggested that some transcripts were probably generated from alternative mRNA splicing or were new transcripts.

**Figure 1:**
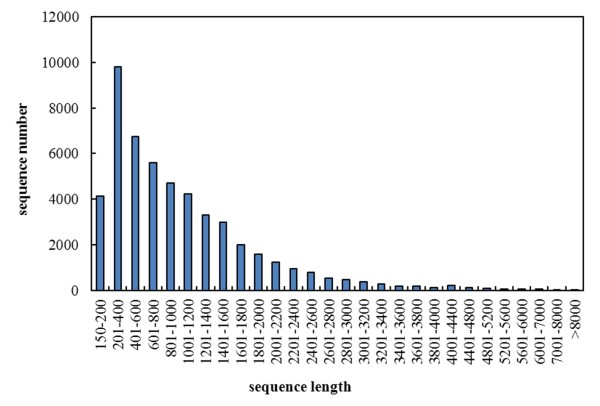
**Length distribution of the transcript sequences detected in the ABA-treated tomato leaf transcriptome.**

The genes and transcripts resulting from Illumina sequencing were merged with the annotated reference genome to generate 37,633 genes and 51,606 transcripts using Cuffmerge [29]. We removed transcripts with lengths less than 150 bp, and the remaining 50,770 transcripts (98.38% of all transcripts), corresponding to 37,093 genes, were used for functional annotation and expression profiling in each sample. Transcripts of length 150–200 bp accounted for 8.14% of the total, those 200–600 bp for 32.53%, those 600–1000 bp for 20.28%, those 1000–1600 bp for 20.72%, those 1600–2200 bp for 9.44%, those 2200–3000 bp for 5.41%, and those >3000 bp for 12.27%, as shown in Figure 1.

### Annotation, functional classification, and KEGG analysis of all detected transcripts

Of the 50,770 transcripts, 45,704 (90.02%) possessed open reading frames (ORF), while ORFs in the other 5,066 (9.98%) were not predicted (Additional file 1: Table S1). Of all transcripts, 47,877 were described in the tomato genome, and 42,583 had homologs in the NCBI non-redundant (NR) protein database. Many of these genes were reported to respond to drought, cold, high salinity, or ABA, and included both regulatory and functional proteins. Transcription factors (1,799 transcripts) were the largest group of regulatory proteins (Additional file 1: Table S1). This result was consistent with the previous reports in *Arabidopsis* and rice using microarray and tiling arrays analysis [20, 23].

**Figure 2:**
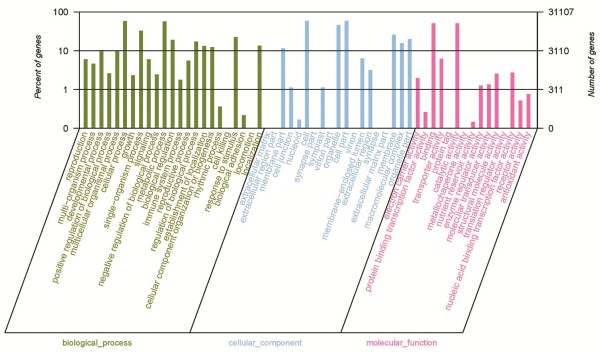
**Gene Ontology (GO) functional annotation of transcripts.** All 31,107 transcripts were assigned to at least one GO term and were grouped into three main GO categories and 57 groups, 23 groups in biological process, 19 in cellular component, and 15 in molecular function. The right-hand Y-axis represents the number of genes in a sub-category. The left-hand Y-axis indicates the percentage of a specific sub-category of genes in each main category.

For the global functional analysis, all identified tomato transcripts were classified into different functional categories using Blast2GO (version 2.3.5) [30]. A total of 31,107 transcripts could be annotated in Gene Ontology (GO) and were classified into 57 functional groups, including 23 groups in biological process, 19 in cellular component, and 15 in molecular function (Figure 2). Within biological process, “metabolic process” (GO: 0008152) with 17,827 transcripts and “cellular process” (GO: 0009987) with 18,169 transcripts were predominant. In the category of cellular component, the three main groups were “cell” (GO: 0005623, 18,493 transcripts), “organelle” (GO: 0043226, 14,423 transcripts) and “cell part” (GO: 0044464, 18,493). The categories “binding” (GO: 0005488) and “catalytic activity” (GO: 0003824) were most common in molecular function, represented by 15,938 and 15,869 transcripts, respectively.

**Figure 3:**
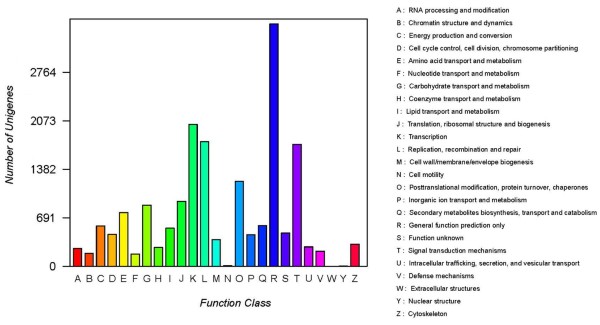
**Function classification in Clusters of Orthologous Groups of proteins (COG).** All transcripts were aligned to the COG database to predict possible functions. A total of 18,885 putative proteins were functionally classified into 25 groups. Capital letters on the X-axis indicate the COG categories as listed on the right of the histogram, and the Y-axis indicates the number of transcripts.

All detected transcripts were blasted to STRING 9.0 for further annotation based on Cluster of Orthologous Groups (COG) of protein categories. A total of 18,885 transcripts with COG annotations were grouped into 25 functional categories. The largest category was “General function prediction only” (3,454 COG annotations, 18.29% of 18885), following by “Transcription” (2,021, 10.70%), “replication, recombination and repair” (1,776, 9.40%), and “signal transduction mechanisms” (1737, 9.20%). In addition, only 474 (2.51%) COG annotations belonged to the “Function unknown” category (Figure 3).

KEGG annotation results were retrieved from KEGG database based on sequence similarity, and 14,371 transcripts were assigned to 310 KEGG pathways. The pathways most strongly represented were “metabolic pathways” (ko01100, 3,476 transcripts), “biosynthesis of secondary metabolites” (ko01110, 1,715), followed by “ribosome” (ko03010, 541), “plant hormone signal transduction” (ko04075, 457), “starch and sucrose metabolism” (ko00500, 267), and “plant-pathogen interaction” (ko04626, 280) (Additional file 1: Table S2).

### Analysis of expressed genes

In total, 39,671 (78.14% of 50,770) transcripts expressed in the tomato leaf transcriptome (Additional file 1: Table S3), including 38,626 in c1d and 37,989 in a1d, respectively. There were 1,682 transcripts expressed only in the ABA-treated group (a1d), 1,045 transcripts expressed only in the control group (c1d), and 36,944 transcripts expressed in both libraries, which indicated that ABA may activate or repress a fraction of unique transcripts.

**Figure 4:**
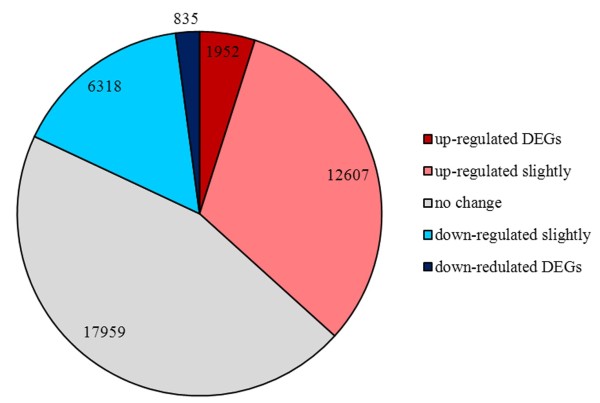
**Differential expression analysis of all transcripts in the control (c1d) and ABA-treatment (a1d) libraries.** Transcripts that differed by less than 20% (|log_2_FC| < 0.25) were assumed to not change in expression level. Transcripts that satisfied the conditions of “FDR < 0.05” and “|log2 fold-change (log_2_FC)| ≥1” were considered significantly differentially-expressed genes (DEGs). Other transcripts are noted as “up-regulated slightly” or “down-regulated slightly”.

Based on expression level, the transcripts were divided into five groups (Table 2). The group with expression levels of 1–10 FPKM (Fragments Per Kilobase of exon model per Million mapped fragments) was largest, representing 44.64% of all transcripts, while those with expression levels of <1 FPKM accounted for 28.87%, those with 10–100 FPKM 23.49%, those with 100–1000 FPKM 2.73%, and those with >1000 FPKM only 0.26%. Among all expressed transcripts, 17,959 showed no changes in expression levels (|log_2_ fold-change (log_2_FC)| < 0.25). The expressions of 21,712 (54.73%) transcripts were altered by exogenous ABA (14,559 were up-regulated, 7,153 were down-regulated) (Additional file 1: Table S3). Of the transcripts with altered expression, 2,787 (12.84% of 21,712) were significantly changed (|log_2_FC| ≥ 1 and false discovery rate (FDR) < 0.05) and labeled differentially expressed genes (DEGs), including 1,952 that were up-regulated and 835 that were down-regulated (Figure 4). Among the DEGs, there were more up-regulated transcripts than down-regulated ones, indicating that many genes responded positively to ABA treatment. This result was similar to those reported in previous studies in *Arabidopsis* and rice [31–33].

**Table 2:** **Number of expressed transcripts in control (c1d) and ABA-treated (a1d) libraries**

FPKM	c1d (control)		a1d (ABA treatment)	
	Number	Percent	Number	Percent
>1000	107	0.28%	90	0.23%
100–1000	1117	2.94%	975	2.52%
10–100	8964	23.60%	9036	23.39%
1–10	16779	44.17%	17428	45.12%
<1	11022	29.01%	11097	28.73%
Total	37989		38626

Molecular functional classification based on GO was also performed on the 2,787 DEGs. The up- and down-regulated DEGs annotated in GO were grouped into 54 and 38 groups on GO.level3, respectively (Additional file 1: Table S4) and into ten classes on GO.level2. The most common categories were “catalytic activity” (654 up-regulated, 295 down-regulated) and “binding” (661 and 260, respectively), followed by “transmembrane transporter activity”, “substrate-specific transporter activity”, “transcription factor activity”, “translation factor activity”, and “signal transducer activity”.

### Genes related to ABA signaling transduction

The ABA signaling transduction pathway mainly includes four core regulatory components: ABA receptor/pyrabactin resistance protein1/PYR-like protein (RCARs/PYR1/PYLs), protein phosphatases type 2C (PP2C), the sucrose nonfermenting1-related protein kinase 2 (SnRK2) and ABA responsive element binding factors (ABF) [34]. In the tomato transcriptome, 18 *PYL*s, 23 *PP2C*s, 12 *SnRK2*s, and 18 *ABF*s were identified (Table 3, Additional file 1: Table S5).

**Table 3:** **Genes related to ABA signaling pathway and transcription factors in tomato transcriptome**

	Description	No. up-regulated	No. down-regulated	No. unchanged	Sum
ABA signaling transduction	PYP/PYL	5	5	8	18
	PP2C	5	8	10	23
	SnRK2	1	4	7	12
	ABF	7	3	8	18
bZIP	ABRT	2	0	2	4
	bZIP	40	21	46	107
BHLH	DREB	2	5	4	11
	BHLH	47	22	66	135
MYB	MYB	66	43	84	193
	MYC	6	2	11	19
AP2	AP2	11	4	11	26
	AP2/ERF	20	14	21	55
	RAV	1	0	2	3
NAC		23	15	25	63
WRKY		33	13	35	81
HSF		16	8	17	41
MADS-box		21	23	17	61

RCARs/PYR1/PYLs, regulatory components of ABA receptor/pyrabactin resistance protein1/PYR-Like proteins; PP2Cs, type 2C protein phosphatases; SnRK2, sucrose nonfermenting1–related protein kinase 2; ABF, ABA responsive element binding factors; ABRT, ABA responsive transcription factor; bZIP, basic leucine zipper transcription factor; DREB, dehydration responsive element binding protein; bHLH, basic helix-loop-helix transcription factor; MYB, myeloblastosis oncogene; MYC, myelocytomatosis oncogene transcription factor; AP2/ERF, apetala 2/ethylene-responsive element-binding factor; NAC, no apical meristem/ATAF/CUP-shaped cotyledons; HSF, heat shock factor.

The discovery of PYR/PYL/RCAR receptors in these samples greatly advances the understanding of the ABA signaling pathway. Among 18 *PYLs* in the tomato transcriptome, the expression level of eight did not differ (|log_2_FC| < 0.25) in ABA treatment compared with control. The other 10 *PYL*s showed slight differences in their transcript abundance, from 0.55 to 1.43 fold, with five up-regulated and five down-regulated genes. Two up-regulated *PYLs* showed moderate transcript level differences (TCONS_00012484, change from 14.16 FPKM in c1d to 18.07 in a1d, TCONS_00036624, from 14.79 to 17.26 FPKM). The other eight differentially-expressed *PYL*s were found at low abundances (FPKM <10). The results indicated that exogenous ABA had a slight impact on *PYL* expression because the ABA-mediated signaling cascade is initiated by the perception of ABA receptors [35]. The slight change in upstream ABA-receptor gene expression could initiate responses in a series of downstream genes. RCARs/PYR1/PYLs were recently found to comprise 14 members in *Arabidopsis thaliana*[6, 36]. All PYL family members are ABA-binding proteins and that regulate the target PP2Cs in a combinatorial manner [37]. However, we detected 18 *PYL* members in this tomato transcriptome, enriching the *PYL* receptor gene family.

Among the 23 *PP2C*s in this study, 13 transcripts changed their expression levels (|log_2_FC| ≥ 0.25), treated by ABA, including five that were up-regulated and eight that were down-regulated. Of them, only one transcript with low levels (TCONS_00014920) was significantly higher than the control by 2.05-fold (from 0.37 to 0.74 FPKM). Three PP2Cs showed significantly decreased expression in ABA-treatment (a1d) by 2.42-fold (TCONS_00014686, from 13.79 to 5.70 FPKM), 3.92-fold (TCONS_00012476 from 16.86 to 4.30 FPKM), and 7.22-fold (TCONS_00030068 from 2.12 to 0.29 FPKM). The result agreed with the previous reports that *PP2C*s were negative regulators of ABA signaling [8]. In the absence of ABA, PYR/PYL/RCARs are not bound to PP2Cs, and PP2C activity is high, which prevents SnRK2 activation. In the presence of ABA, the combination of ABA and PYR/PYL/RCARs bind to and inhibit PP2Cs, which allows the accumulation of phosphorylated SnRK2s and subsequent phosphorylation of ABFs [8].

Among the 12 *SnRK2* transcripts, five differed slightly in level compared with control. Of them, one showed increased expression (TCONS_00011840 from 4.41 to 5.34 FPKM) and four showed decreased expression (TCONS_00017720, TCONS_00017721, TCONS_00044604, TCONS_00046427). Although TCONS_00017721 had moderate transcript abundance (FPKM from 40.56 to 26.80 FPKM), the other three down-regulated *SnRK2*s were expressed at low levels (FPKM < 1.04). SnRK2s are categorized into subclasses I, II and III. Subclass I members are not activated by ABA, and subclass II and III members are weakly and strongly activated by ABA, respectively [38]. Among 10 *SnRK2* members in *Arabidopsis* and rice, *SAPK8*, *SAPK9,* and *SAPK10* were in subclass III, and *SAPK1* to *SAPK7* were in subclass I and II [38]. In this transcriptome, the only induced *SnRK2* (TCONS_00011840) that was annotated as serine/threonine protein kinase *SAPK8*-like protein was of subclass III. The other four repressed *SnRK2*s were possibly *SAPK1*, *SAPK3*, or *SAPK7* based on their NCBI blastx matches. Kobayashi et al. (2004) demonstrated that only three *SnRK2* family members were activated by ABA but all were activated by hyperosmotic stress, and there were no members activated only by ABA. This study also suggested that a small percentage of *SnRK2*s participated in the signaling transduction under exogenous ABA stress.

In the 18 *ABF*s, seven were up-regulated and three were down-regulated. TCONS_00038922 (from 0.16 to 0.43 FPKM) and TCONS_00038921 (from 0.43 to 0.10 FPKM) were significantly altered. This result indicated that *ABF*s were mainly positive regulators of ABA response. The members of the ABF gene family belongs to a subfamily of *bZIP* TFs [6]. Most *ABF*s are known induced in vegetative tissue under drought stress and ABA treatment in *Arabidopsis*[9]. However, in the transcriptome, *ABF*s were expressed at low levels in both libraries, so transcription regulator factors may target a cascade action [39].

Overall, these results agreed with what is known of the ABA regulation pathway [6, 8, 40]. Thus exogenous ABA can activate the signaling pathway, and it affected *PYL*s, *PP2C*s and *SnRK2*s, and *ABF*s. Moreover, members of these gene families had different responses to ABA, indicating that they probably played positive or negative regulatory roles in the ABA pathway. Furthermore, many genes in these families had no differences in expression abundance, implying that there exists functional redundancy in the ABA pathway or that these genes participate in an ABA-independent pathway [8].

### Transcription factors induced by ABA

With respect to TFs, the results of this study supported those of previous research. The TF families of *bZIP* (111 transcripts), *bHLH* (146), *MYB* (212), *AP2/ERF* (84), *NAC* (63), and *WRKY* (81) were identified in this study. Moreover, 41 *Heat shock factors* (*HSF*s) and 61 *MADS-boxes* were also detected (Table 3). In the *bZIP* family, *ABA-responsive TF* (four transcripts) and *bZIP* (107 transcripts) were two subgroups. Dehydration-responsive element-binding protein (*DREB*, 11) and *bHLH* (135) belonged to the *bHLH* family. The *MYB* family included *MYB* (193) and *MYC* (19). The *AP2* family was divided into three subgroups, including *AP2* (26 transcripts), *AP2/ERF* domain-containing TF (55), and *RAV1* (3). Reportedly, the most common classes of regulatory sequences induced by ABA are the *G-box ABREs* recognized by members of the *bZIP* family, and many ABA-regulated genes also contain binding sites for proteins of the *MYB* and *MYC* families. *NAC*, *WRKY*, *bHLH*, and Zn-finger classes were also found to participate in some ABA responses [8].

Although TFs were up-regulated after ABA treatment, they had low abundances in both libraries (FPKM < 10) (Table 3, Additional file 1: Table S5). The TFs interacted with cis-elements in the promoter regions of several responsive genes and thus control the expressions of many downstream genes, triggering cascade reactions of many physiological processes and controlling biochemical reactions in plant cells [39]. Thus, a slight alteration in the transcript abundance of TFs can result in a substantial change in downstream gene expression and physiological responses. Therefore, although in this tomato transcriptome ABA affected TFs with low transcript abundances, the potential results cannot be neglected. Exceptionally, dehydration-responsive element binding proteins (*DREB*s) that bind to drought-response elements (DRE) cis-elements were substantially down-regulated (five down-regulated and two up-regulated). Reportedly, most *DREB*s are independent of ABA [41].

### Genes related to heat shock protein (HSP)

Heat shock proteins (HSPs), including HSP70s, HSP90s, HSP100s, HSP60s (cpn60s), and small heat-shock proteins (sHSPs), are stress-responsive proteins that function as molecular chaperones, protecting plants from damage under stress [42]. In the tomato transcriptome, 83 transcripts were identified as *HSP*s, including 12 *HSP90s*, 36 *HSP70*s and 24 *sHSP*s, mainly *HSP20*s, as well as 11 other *HSP* transcripts (Table 4, Additional file 1: Table S5).

**Table 4:** **Genes related to heat shock proteins (HSPs), reactive oxygen species (ROS) scavenging, and pathogens resistance**

Trait		Description	No. of up-regulated	No. of down-regulated	No. of no change	Sum
Heat shock proteins		Hsp90	3	1	8	12
		HSP70	8	8	20	36
		sHSP	4	11	9	24
		Other HSP	2	0	9	12
		SOD	1	1	8	10
		CAT	5	0	2	7
ROS scavenging system	GSH-AsA cycle	GLR	7	16	12	35
		APX	4	3	13	20
		MDAR	1	0	4	5
		DHAR	2	1	5	8
		GR	1	0	3	4
	GPX pathway	GST	23	20	33	76
		GPX	0	1	7	8
		POD	33	28	31	92
	PrxR/Trx pathway	Trx	25	29	79	133
		PrxR	1	5	4	10
pathogens resistance	Some proteins	PAL	6	3	7	16
		PPO	4	0	1	5
		GLU	14	12	36	62
		chitinase	15	4	17	36
	SA signaling	TGA	10	2	14	26
		PR1	15	12	18	45
	JA signaling	JAR1	7	2	6	15
		JAZ	6	6	6	18
	ET signaling	ETR	6	5	3	14
		ERF/EREBP	5	4	6	15

APX, ascorbate peroxidase; CAT, catalase; DHAR, dehydroascorbate reductase; ERF/EREBP, ethylene response factor; ET, ethylene; ETR, ethylene receptor; GLR, glutaredoxin; GLU, beta-1,3-glucanase; GPX, glutathione peroxidase; GR, glutathione reductase; GSH-AsA, glutathione-ascorbate; GST, glutathione-S-transferase; HSP, heat shock protein; JA, jasmonic acid; JAR1, jasmonate resistant 1; JAZ, jasmonate ZIM-domain protein; MDAR, monodehydroascorbate reductase; PAL, phenylalanine ammonia-lyase; POD, peroxidase; PPO, polyphenol oxidase; PR1, pathogenesis-related proteins; PrxR, peroxiredoxin; ROS, reactive oxygen species; SA, salicylic acid; sHSP, small heat shock protein; SOD, superoxide, TGA, TGACG/as-1, binding; Trx, Thioredoxin.

HSP90s and HSP70s are important members of the HSP protein family. The major role of HSP90 is to manage protein folding [43, 44], and it also plays key roles in signal-transduction networks, cell-cycle control, protein degradation, and protein trafficking [45]. HSP70s function in preventing aggregation, assisting refolding, protein import and translocation, signal transduction, and transcriptional activation [42, 46]. In this transcriptome, three up-regulated and one down-regulated *HSP90*s were weakly expressed. Two of the three up-regulated *HSP90*s had high transcript abundances (TCONS_00021180 from 95.04 to 110.50 FPKM, TCONS_00032910 from 868.34 to 966.19 FPKM), while the down-regulated *HSP90* was expressed at low abundance. Among 36 *HSP70*s, 16 transcripts differed in expression level, with eight up-regulated and eight down-regulated, although the differences in expression were slight. The up-regulated TCONS_00037214 (from 989.52 to 1116.60 FPKM) and the down-regulated TCONS_00017664 (from 105.12 to 80.45 FPKM) showed high and moderate transcript abundances, respectively. The other changed *HSP70*s were expressed at low abundances.

Although many *HSP70*s and *HSP90*s were slightly up-regulated, some showed high transcript abundances. For a highly-abundant transcript, a slightly changed ratio may substantially alter its abundance and induce physiological changes. This research indicated that *HSP70* and *HSP90* may play an important role in ABA responses.

Small HSPs are a large family present ubiquitously in all organisms. Plants have many more sHSPs than other eukaryotes [47]. In 24 *sHSP*s, 15 slightly changed their expression levels by ABA, including four up-regulated and 11 down-regulated transcripts. All up-regulated *sHSP*s had low transcript abundances in both the ABA treatment and control. Among the down-regulated *sHSP*s, there were five with moderate abundance (10 < FPKM < 100) in both libraries (TCONS_00036382, TCONS_00026922, TCONS_00028974, TCONS_00035725, TCONS_00023776), and the other six had low abundances. sHSPs bind denatured proteins and prevent their irreversible aggregation, so they are referred to as ‘paramedics of the cell’ [48]. Therefore, they have a crucial and fundamental role in plant cell biology [47, 48], and contribute to the molecular adaptation to a wide range of environmental stresses, including heat, cold, drought, salinity, and oxidative stress [42].

At present, studies of ABA responses to HSP are limited. *HSP101* mRNAs in wheat leaves were reported to be induced by 2 h of dehydration and 50 μM ABA [49]. In an examination of nine rice *HSP* genes under abiotic stresses and ABA treatment, only *OsHSP71.1* was induced by ABA while *OsHSP24.1* was suppressed [50]. In this study, several *HSP70*s and *HSP90*s were up-regulated, while most *sHSP*s were down-regulated. These observations imply that the *HSP* genes may play different roles in plant development and abiotic stress responses. Furthermore, the relationship between ABA and HSPs will be interesting to investigate in the future.

### Genes related to the reactive oxygen species (ROS) scavenging system

Overproduction of reactive oxygen species (ROS) and overexpression of various antioxidant enzymes in the ROS scavenging system occur during almost all biotic and abiotic stresses [51]. In total, there were 408 transcripts identified as encoding enzymes in the ROS scavenging system (Table 4, Additional file 1: Table S5). They were categorized into the glutathione-ascorbate (GSH-AsA) cycle (72 transcripts), the glutathione peroxidase (GPX) pathway (176), the peroxiredoxin/thioredoxin (PrxR/Trx) pathway (143), superoxide dismutase (SODs, 10) and the catalases (CATs, 7). The largest families were genes encoding thioredoxins (Trxs, 133 transcripts), peroxidases (PODs, 92), and glutathione S-transferases (GSTs, 76).

In a stress environment, the first step in O_2_ reduction produces diffusible hydroperoxyl (HO_2_^−^) and superoxide (O_2_^−^) radicals [52]. Meanwhile, SODs are induced to rapidly disproportionate O_2_^−^ into oxygen and H_2_O_2_. However, of nine *SOD*s, one with high abundance (TCONS_00025638, from 204.01 to 265.26 FPKM) was slightly down-regulated, and another (TCONS_00025639, from 0 to 0.012 FPKM) showed slightly increased expression level. The other six *SOD*s showed no changes (|log_2_FC| < 0.25). It was also found that the expression levels of SODs showed slightly change after ABA treatment in Arabidopsis [23].

CAT reduces H_2_O_2_ directly to water and oxygen. In this study, the most of the genes encoding CAT were up-regulated by ABA, suggesting the importance of CATs in scavenging ROS under ABA stress. Among the seven identified *CAT* genes (five up-regulated, two unchanged), the abundances of TCONS_00003149, TCONS_00003243, TCONS_00049411, TCONS_00051177, and TCONS_00003244 in ABA treatment were higher than in control by 3.33, 2.20, 1.52, 1.45, and 1.23 fold, respectively. In particular, TCONS_00049411 (from 115.88 to 176.01 FPKM) and TCONS_00051177 (from 91.12 to 131.86 FPKM) had high abundance, and TCONS_00003243 was expressed a moderate levels (from 9.43 to 20.70 FPKM).

Moreover, H_2_O_2_ can be also indirectly scavenged by the ascorbate-glutathione (AsA-GSH) cycle, the GPX pathway, and the PrxR/Trx pathway [53, 54]. The AsA-GSH cycle contained 35 glutaredoxins (*GLR*s, seven up-regulated, 16 down-regulated), 20 ascorbate peroxidases (*APX*s, four up-regulated, three down-regulated), five monodehydroascorbate reductases (*MDAH*s, one up-regulated, four unchanged), eight dehydroascorbate reductases (*DHAR*s, two up-regulated, one down-regulated), and four glutathione reductases (*GR*s, one up-regulated, three unchanged). Most transcripts showed slight differences in their expression levels between the two libraries. Only three transcripts, TCONS_00040877 (*GLR*, from 3.77 to 8.24 FPKM), TCONS_00036965 (*APX*, from 0.00020 to 1.83 FPKM) and TCONS_00020530 (*DHAR*, from 0.28 to 1.44 FPKM) were significantly up-regulated.

In the GPX pathway, there were 76 *GST*s (23 up-regulated, 20 down-regulated), eight glutathione peroxidases (*GPX*s, one down-regulated, seven unchanged), and 92 *POD*s (33 up-regulated, 28 down-regulated). All up-regulated genes had low transcript abundances except TCONS_00018678 (*POD*), which was moderately abundant. Most down-regulated transcripts were expressed at low abundance, six occurred at moderate abundance (3 *GST*s: TCONS_00037287, TCONS_00037288, and TCONS_00037289; one *GPX*: TCONS_00034706 and 3 *POD*s: TCONS_00018679, TCONS_00009154, and TCONS_00004157), and two *GST*s (TCONS_00025295 and TCONS_00039081) were highly abundant. Moreover, most transcripts in this group showed slight alterations in expression level between the libraries. Exceptionally, one *GST* (TCONS_00005473) and three *POD*s (TCONS_00046741, TCONS_00004986, and TCONS_00003494) were significantly up-regulated and three *GST*s (TCONS_00008673, TCONS_00005786, and TCONS_00025296) and two *POD*s (TCONS_00003495 and TCONS_00009061) were significantly down-regulated.

There were 133 transcripts encoding Trxs (25 up-regulated, 29 down-regulated) and 10 transcripts encoding PrxRs (one up-regulated, five down-regulated) in the PrxR/Trx pathway. All up-regulated *Trx*s were expressed at low abundance in both libraries (FPKM < 10), except TCONS_00023279 and TCONS_00024016. Among the down-regulated *PrxR* and *Trx* genes, more than half were moderately or highly abundant.

The results of this study showed that most transcripts of *APX*, *MDAR*, *DHAR*, *GR*, *GPX*, *GLR*, *PrxR*, and *Trx* were down-regulated or did not change in expression level. These enzymes reduce oxidative damage by H_2_O_2_ in cells directly or indirectly, depending on the GSH redox state, which is catalyzed by GR [54–56]. Previous research demonstrated that H_2_O_2_ and the GSH/GSSG couple may interact with other signaling pathways that regulate the expression of antioxidant genes during stress responses [57]. However, this study showed that exogenous ABA repressed the expression of the genes encoding enzymes related to the GSH/GSSG couple, suggesting that ABA signaling may be independent of GSH redox signaling.

Furthermore, more than half of the *GST*s, which may catalyze the conjugation reaction of GSH and protect SH-containing enzymes against oxidation, were induced by ABA [58]. ABA probably also caused the down-regulation of genes encoding enzymes that depend on GSH. POD catalyzes an early step in lignin synthesis in the phenylalanine metabolism pathway, which can increase the rigidity of cell walls to protect plants from pathogenic bacteria [59] and allowing them to resist wilting and mechanical weakness imparted by abiotic stresses [60]. This study found 33 ABA-induced *POD* genes that maybe participated in these processes.

### Genes related to pathogen resistance

The phytohormone ABA plays a multifaceted and pivotal role in plant immunity. In the whole transcriptome, a large number of genes related to disease resistance were detected, including 16 *PAL* transcripts (six up-regulated, three down-regulated), five *polyphenol oxidase* (*PPO*) transcripts (four up-regulated, one unchanged), 62 *beta-1,3-glucanase* (*GLU*) transcripts (14 up-regulated, 12 down-regulated), and 36 *chitinase* transcripts (15 up-regulated and four down-regulated). There were more up-regulated isoforms than down-regulated ones, and the expression changes of up-regulated isoforms were greater than down-regulated one. The results suggested that ABA has a greater positive than negative impact on tomato pathogen resistance.

The four up-regulated *PAL*s (TCONS_00038804, TCONS_00037017, TCONS_00038803, and TCONS_00013292), the up-regulated *PPO* (TCONS_00036090), the up-regulated *chitinase* (TCONS_00024439), and one down-regulated *GLU* (TCONS_00032191) showed high or moderate abundances. Other transcripts of *PAL*, *PPO*, *GLU*, and *chitinase* genes were expressed at low abundance, including the significantly up-regulated *PPO* (TCONS_00036092), *GLU*s (TCONS_00045778 and TCONS_00048495), and *chitinase* (TCONS_00011298) (Table 4, Additional file 1: Table S5). *PAL*, *PPO*, *GLU* and *chitinase* are often associated with improved plant resistance to pathogens [61–63]. Our previous study showed that foliar spraying of exogenous ABA (7.58 μmol L^-1^) could enhance resistance to *Alternaria solani* (early blight) infection in tomato by activating defense genes and enhancing the activities of defense-related enzymes, including *PAL*, *PPO* and *POD*, *PR1*, *GLU*, and *SOD*[17].

In this transcriptome, many genes in the salicylic acid (SA), jasmonic acid (JA), and ethylene (ET) signal transduction pathways were detected. These pathways play well-established roles in phytopathogen defenses [64]. TGACG/as-1 bindings (TGAs, 26 transcripts in this study) and pathogenesis-related proteins (PR1s, 45 transcripts) are critical component in the SA signaling pathway, while jasmonate resistant 1 (JAR1s, 15 transcripts) and jasmonate ZIM-domain proteins (JAZs, 18 transcripts) are crucial in the JA signaling pathway and ethylene receptors (ETRs, 14 transcripts) and ethylene response factor/ethylene-responsive TFs (ERF/EREBPs, 15 transcripts) are vital to the ET signaling pathway (Table 4, Additional file 1: Table S5). Most of these genes were up-regulated by exogenous ABA and most were expressed at low abundance.

Salicylic acid, a key phytohormone in plant–pathogen interactions, is typically associated with systemic expression of pathogenesis-related protein genes against biotrophs and hemibiotrophs. This response often culminates in a localized cell death termed the hypersensitive response [65–67]. JA and ET predominantly protected *Arabidopsis* against biotrophic pathogens [68]. In tomato, the comparative transcriptome results indicated that ABA played a positive role in biotic stress resulting from the crosstalk between ABA and SA, JA, and ET.

In general, ABA is thought to play a negative role in plant resistance to both biotrophic and necrotrophic fungi and bacteria, and ABA interacts antagonistically with SA, JA, and ET in various ways [69]. For example, in tomato, the ABA-deficient mutant *sitiens* was much more resistant to *Erwinia chrysanthemi*, and exogenous ABA (100 μM) made both the wild type and *sitiens* more susceptible to *E. chrysanthemi*[18, 70]. ABA (100 μM) decreased the disease resistance of rice to rice blast (*Magnaporthe grisea*) by suppressing some genes in the SA signaling pathway [71]. However, in this study, ABA largely up-regulated the genes related to biotic stresses, indicating that ABA has potential to promote pathogen resistance in tomato. Furthermore, the concentration of ABA applied here (7.58 μM) was much lower than that used in most previous reports (usually 100 μM). We hypothesize that the ABA concentration is very important in determining whether plant resistance to pathogens will be improved or suppressed.

ABA not only mediates tolerance to adverse environmental conditions, but also affects the resistance to biotic stresses. It plays critical roles in the interrelationship between biotic and abiotic stress responses. Further insights into the core network of ABA signaling in plants will be crucial for genetically engineering and breeding crop species with both improved abiotic stress tolerance and pathogen resistance [72]. ABA has also been recognized to crosstalk with the “developmental” hormones auxin, gibberellic acid, cytokinins, and brassinosteroids in plant immunity [14, 15, 69]. In this study, the expressions of many genes related to these hormones were also affected by ABA (Additional file 1: Table S1). These genes may directly or indirectly contribute to plant pathogen resistance.

### Quantitative real-time-PCR validation of differentially-expressed transcripts from RNA-seq

To confirm the accuracy and reproducibility of this Illumina RNA-seq result, 25 transcripts were chosen randomly for quantitative real-time (qRT) PCR. Those genes were involved in metabolism, information transfer, or were hypothetical proteins, and included up-regulated, down-regulated, and unaffected transcripts. The primer sequences, gene functions, and FPKM and qRT-PCR values are listed in Additional file 1: Table S6. The qRT-PCR results generally agreed (84%) with the changes in transcript abundance determined by RNA-seq, suggesting the reliability of the RNA-seq data.

## Conclusion

Genome-wide transcriptome analysis results indicated that exogenous ABA can influence the ABA signaling pathway with the core of *PYR/PYL/RCARs-PP2Cs-SnRK2s.* Exogenous ABA up-regulated many genes related to stress tolerance and pathogen resistance, including various TFs, *HSP90*s, *HSP70*s, *CAT*s, *GST*s, *POD*s, *PAL*s, *PPO*s and *chitinases*, as well as the genes *TGA*, *PR1*, *JAR1*, *JAZ*, *ERT* and *ERF/EREBP* in the SA, JA, and ET signaling pathways. These results suggested that ABA has the potential to improve the abiotic and biotic tolerance of tomato. The study extends the knowledge of the complex molecular and cellular events during ABA signaling and of ABA-induced genes. Challenges and opportunities remain in exploring the complex interactions between ABA and defense in whole plants.

## Methods

### Plant material for RNA-Seq

Seeds of tomato cv. Hongtaiyang 903 were planted and grown in plastic pots filled with organic loam in April, 2012, and grown in a glasshouse in Chengdu, Sichuan Province, China. Sixty pots (four seedlings per pot) were used in this experiment. They were watered every other day. After 45 days when the plants had 5–7 leaves, the 60 pots were divided into two groups. The plants in 30 pots were sprayed with 400 mL of 7.58 μmol L^-1^ ABA solution, and the plants in the other 30 pots were sprayed with the same volume of purified water as a control. Twenty-four hours later, the young third leaves of ten randomly-selected plants from both the ABA-treated and control groups were collected. The leaves of each group were combined, immediately snap-frozen, and stored in liquid nitrogen. These samples were labeled a1d (1 d after ABA-treatment) and c1d (1 d, control).

### RNA extraction, cDNA library construction and Illumina deep sequencing

Total RNA samples of a1d and c1d were prepared using Trizol reagent (Invitrogen, Carlsbad, CA, USA) and subsequently used for mRNA purification and library construction with the Truseq™ RNA Sample Prep Kit (Illumina, San Diego, CA, USA) following the manufacturer’s instructions. The two samples were sequenced on an Illumina HiSeq™ 2000 (Illumina), generating 165,894,496 reads. Each sample yielded more than 12 Gb of data. Sequencing was completed by the Shanghai Majorbio Bio-pharm Biotechnology Co. (Shanghai, China).

### Read trimming and optimization

For each set of sequencing reads, the sequencing adapters were trimmed using SeqPrep (https://github.com/jstjohn/SeqPrep), and then low quality bases (Solexa/Illumina quality score < 25) of the 3′ ends were trimmed using in-house Perl scripts. The remaining high-quality reads were submitted for mapping analysis against the reference genome sequences (ftp://ftp.solgenomics.net/tomato_genome/annotation/ITAG2.3_release/) using Tophat [73]. The mapped reads were then assembled with Cufflink [29]. The assembled results and original genome annotations were merged and used for further annotation and differential-expression analysis.

All of these RNA-Seq reads were deposited in Sequence Read Archive database (http://www.ncbi.nlm.nih.gov/Traces/sra/) under the Accession number of SRR926182 and SRR926185.

### Mapping reads to the reference genome and annotated genes

Open reading frames (ORFs in all transcripts were predicted using Trinity (http://trinityrnaseq.sourceforge.net/analysis/extract_proteins_from_trinity_transcripts.html) [74]. Sequence-similarity Blast searches of these transcripts were conducted against the tomato genome reference, the NCBI NR protein database (http://www.ncbi.nlm.nih.gov/), the Gene Ontology (GO) database (http://www.geneontology.org/), the Search Tool for the Retrieval of Interacting Genes (STING) database (http://string-db.org/) [75], and the Kyoto Encyclopedia of Genes and Genomes (KEGG) database (http://www.genome.jp/kegg/) [76]. GO terms for tomato transcripts were obtained using Blast2GO (v. 2.3.5) (http://www.blast2go.org/) with default parameters [30]. COG terms were obtained using Blastx 2.2.24+ in STRING 9.0. Metabolic pathway analysis was performed using Blastx/Blastp 2.2.24+ in KEGG (http://www.genome.jp/kegg/genes.html).

### Differential expression analysis

The Tophat (http://tophat.cbcb.umd.edu/) and Cufflinks (http://cufflinks.cbcb.umd.edu/) programs provide FPKM (Fragments Per Kilobase of exon model per Million mapped fragments) values within a 95% confidence interval. Differential expression was analyzed and calculated according to the count values of each transcript in the two libraries using edgeR (the Empirical analysis of Digital Gene Expression in R) software [77]. “FDR < 0.05” and “|log_2_ fold-change (log_2_FC)| ≥1” were used as the thresholds for judging significant difference in transcript expression. Transcripts with |log_2_FC| < 0.25 were assumed have no change in expression levels.

### qRT-PCR verification

qRT-PCR was performed to verify the expression patterns revealed by the RNA-seq study. The purified RNA samples were reverse-transcribed using the PrimeScript RT Reagent Kit with gDNA Eraser (Takara, Dalian, China) following the manufacturer’s protocol. Twenty-five transcripts were selected randomly for the qRT–PCR assay. Gene specific qRT–PCR primers (18–20 bp) (Additional file 1: Table S6) were designed using Premier 5.0 software (Premier Biosoft International, Palo Alto, CA). qRT–PCR was performed using SybrGreen qRT-PCR Master Mix (Ruian Biotechnologies, Shanghai, China) in an ABI 7500 FAST Real-Time PCR System (Applied Biosystems, Foster City, CA, USA). PCR conditions were 2 min at 95°C, followed by 40 cycles of heating at 95°C for 10 s and annealing at 60°C for 40 s. Three replicates were performed, and the amplicons were used for melting curve analysis to check the amplification specificity. The relative expression level of each gene was calculated as 2^-(∆∆Ct)^ and the housekeeping gene *glyceraldehyde-3-phosphate dehydrogenase* gene (Accession No. U93208) from *S. lycopersicum* was used to normalize the amount of template cDNA added in each reaction.

## Electronic supplementary material

Additional file 1: Table S1: Annotation of transcripts according to the NCBI non-redundant, Gene Ontology, and STRING databases and the tomato genome. **Table S2.** KEGG pathway mapping. **Table S3.** Differently-expressed transcripts in the control (c1d) and ABA-treatment (a1d) libraries. **Table S4.** Molecular functional classification of the significantly differentially-expressed genes (DEGs) based on GO.level2 and GO.level3. **Table S5.** Genes related to ABA signaling transduction, transcription factors, heat shock proteins (HSPs), reactive oxygen scavenging enzymes, and pathogen resistance. **Table S6.** Comparison of expression patterns between RNA-seq expression and quantitative real-time PCR. (XLSX 12 MB)

## Abbreviations

ABA: Abscisic acid
a1d: ABA-treatment after 1 day
ABF: ABA responsive element (ABRE) binding factors
ABRE: ABA responsive promoter element
ABRT: ABA responsive transcription factor
ANT: ADP/ATP translocator-like
AP2/ERF: apetala 2/ethylene -responsive element-binding factor
APX: Ascorbate peroxidase
AREB: ABA-responsive element binding protein
bHLH: Basic helix-loop-helix
bZIP: Basic leucine zipper
c1d: Control sample after 1 day
CAT: Catalase
cDNA: Complementary DNA (deoxyribonucleic acid)
COG: Clusters of orthologous groups of proteins
DEG: Significantly differentially expressed genes
DHAR: Dehydroascorbate reductase
DREB: Dehydration responsive element binding protein
ERF/EREBP: Ethylene response factor
ET: ethylene
ETR: ethylene receptor
FPKM: Fragments Per Kilobase of exon model per Million mapped fragments
GAPDH: Glyceraldehyde-3-phosphate dehydrogenase
GLR: Glutaredoxin
GLU: Beta-1:3-glucanase
GO: Gene ontology
GPX: Glutathione peroxidase
GR: Glutathione reductase
GSH-AsA: glutathione-ascorbate
GST: Glutathione-S-transferase
HSF: Heat shock factor
HSP: Heat shock protein
JA: Jasmonic acid
JAR1: Jasmonate resistant
JAZ: Jasmonate ZIM-domain protein
KEGG: Kyoto Encyclopedia of genes and genomes
MDAR: Monodehydroascorbate reductase
MYB: Myeloblastosis oncogene
MYC: Myelocytomatosis oncogene
NAC: No apical meristem/ATAF/CUP-shaped cotyledons
NATs: Natural antisense transcripts
NR: Non-redundant protein database in National Center of Biotechnology Information (NCBI)
ORF: Open reading frame
PAL: Phenylalanine ammonia-lyase
PCR: Polymerase chain reaction
POD: Peroxidase
PP2Cs: Protein phosphatases which fall under the category of type 2C
PPO: Polyphenol oxidase
PR1: Pathogenesis-related proteins
PrxR: Peroxiredoxin
RCARs/PYR1/PYL: Regulatory components of ABA receptor/pyrabactin resistance protein1/PYR-like protein
RNA: Ribonucleic acid
ROS: Reactive oxygen species
qRT-PCR: Quantitative real time - PCR
SA: Salicylic acid
sHSP: Small heat shock protein
SnRK2: The sucrose nonfermenting1–related protein kinase 2
SOD: Superoxide
STING: Search tool for the retrieval of interacting genes
TGA: TGACG/as-1: binding
Trx: Thioredoxin.

## References

[1] Gupta S, Shi X, Lindquist IE, Devitt N, Mudge J, Rashotte AM (2013). Transcriptome profiling of cytokinin and auxin regulation in tomato root. J Exp Bot.

[2] Bonierbale MW, Plaisted RL, Tanksley SD (1988). RFLP maps based on a common set of clones reveal modes of chromosomal evolution in potato and tomato. Genetics.

[3] Sato S, Tabata S, Hirakawa H, Asamizu E, Shirasawa K, Isobe S, Kaneko T, Nakamura Y, Shibata D, Aoki K (2012). The tomato genome sequence provides insights into fleshy fruit evolution. Nature.

[4] Finkelstein RR, Gampala SSL, Rock CD (2002). Abscisic acid signaling in seeds and seedlings. Plant Cell.

[5] Parent B, Hachez C, Redondo E, Simonneau T, Chaumont F, Tardieu F (2009). Drought and abscisic acid effects on aquaporin content translate into changes in hydraulic conductivity and leaf growth rate: a trans-scale approach. Plant Physiol.

[6] Raghavendra AS, Gonugunta VK, Christmann A, Grill E (2010). ABA perception and signalling. Trends Plant Sci.

[7] Chinnusamy V, Gong ZZ, Zhu JK (2008). Abscisic acid-mediated epigenetic processes in plant development and stress responses. J Integr Plant Biol.

[8] Cutler SR, Rodriguez PL, Finkelstein RR, Abrams SR (2010). Abscisic acid: emergence of a core signaling network. Annu Rev Plant Physiol Plant Mol Biol.

[9] Fujita Y, Fujita M, Shinozaki K, Yamaguchi-Shinozaki K (2011). ABA-mediated transcriptional regulation in response to osmotic stress in plants. J Plant Res.

[10] Wang RS, Pandey S, Li S, Gookin TE, Zhao Z, Albert R, Assmann SM (2011). Common and unique elements of the ABA-regulated transcriptome of Arabidopsis guard cells. BMC Genomics.

[11] Kumar S, Kaur G, Nayyar H (2008). Exogenous application of abscisic acid improves cold tolerance in chickpea (cicer arietinum L.). J Agron Crop Sci.

[12] Kumar S, Kaushal N, Nayyar H, Gaur P (2012). Abscisic acid induces heat tolerance in chickpea (cicer arietinum L.) seedlings by facilitated accumulation of osmoprotectants. Acta Physiol Plant.

[13] Gurmani AR, Bano A, Khan SU, Din J, Zhang JL (2011). Alleviation of salt stress by seed treatment with abscisic acid (ABA), 6-benzylaminopurine (BA) and chlormequat chloride (CCC) optimizes ion and organic matter accumulation and increases yield of rice (Oryza sativa L.). Aust J Crop Sci.

[14] Grant MR, Jones JDG (2009). Hormone (Dis)harmony moulds plant health and disease. Science.

[15] Mauch-Mani B, Mauch F (2005). The role of abscisic acid in plant-pathogen interactions. Curr Opin Plant Biol.

[16] Audenaert K, De Meyer GB, Hofte MM (2002). Abscisic acid determines basal susceptibility of tomato to Botrytis cinerea and suppresses salicylic acid-dependent signaling mechanisms. Plant Physiol.

[17] Song W, Ma X, Tan H, Zhou J (2011). Abscisic acid enhances resistance to Alternaria solani in tomato seedlings. Plant Physiol Biochem.

[18] Asselbergh B, De Vleesschauwer D, Hofte M (2008). Global switches and fine-tuning - ABA modulates plant pathogen defense. Mol Plant Microbe In.

[19] Ton J, Flors V, Mauch-Mani B (2009). The multifaceted role of ABA in disease resistance. Trends Plant Sci.

[20] Rabbani MA, Maruyama K, Abe H, Khan MA, Katsura K, Ito Y, Yoshiwara K, Seki M, Shinozaki K, Yamaguchi-Shinozaki K (2003). Monitoring expression profiles of rice genes under cold, drought, and high-salinity stresses and abscisic acid application using cDNA microarray and RNA gel-blot analyses. Plant Physiol.

[21] Seki M, Ishida J, Narusaka M, Fujita M, Nanjo T, Umezawa T, Kamiya A, Nakajima M, Enju A, Sakurai T (2002). Monitoring the expression pattern of around 7,000 Arabidopsis genes under ABA treatments using a full-length cDNA microarray. Funct Integr Genom.

[22] Buchanan CD, Lim SY, Salzman RA, Kagiampakis L, Morishige DT, Weers BD, Klein RR, Pratt LH, Cordonnier-Pratt MM, Klein PE (2005). Sorghum bicolor’s transeriptome response to dehydration, high salinity and ABA. Plant Mol Biol.

[23] Matsui A, Ishida J, Morosawa T, Mochizuki Y, Kaminuma E, Endo TA, Okamoto M, Nambara E, Nakajima M, Kawashima M (2008). Arabidopsis transcriptome analysis under drought, cold, high-salinity and ABA treatment conditions using a tiling array. Plant Cell Physiol.

[24] Nemhauser JL, Hong F, Chory J (2006). Different plant hormones regulate similar processes through largely nonoverlapping transcriptional responses. Cell.

[25] Nie QH, Fang MX, Jia XZ, Zhang W, Zhou XN, He XM, Zhang XQ (2011). Analysis of muscle and ovary transcriptome of Sus scrofa: assembly: assembly, annotation and marker discovery. DNA Res.

[26] Cloonan N, Forrest ARR, Kolle G, Gardiner BBA, Faulkner GJ, Brown MK, Taylor DF, Steptoe AL, Wani S, Bethel G (2008). Stem cell transcriptome profiling via massive-scale mRNA sequencing. Nat Methods.

[27] Nagalakshmi U, Wang Z, Waern K, Shou C, Raha D, Gerstein M, Snyder M (2008). The transcriptional landscape of the yeast genome defined by RNA sequencing. Science.

[28] Hermans C, Vuylsteke M, Coppens F, Craciun A, Inze D, Verbruggen N (2010). Early transcriptomic changes induced by magnesium deficiency in Arabidopsis thaliana reveal the alteration of circadian clock gene expression in roots and the triggering of abscisic acid-responsive genes. New Phytol.

[29] Trapnell C, Williams BA, Pertea G, Mortazavi A, Kwan G, van Baren MJ, Salzberg SL, Wold BJ, Pachter L (2010). Transcript assembly and quantification by RNA-Seq reveals unannotated transcripts and isoform switching during cell differentiation. Nat Biotechnol.

[30] Conesa A, Götz S, García-Gómez JM, Terol J, Talón M, Robles M (2005). Blast2GO: a universal tool for annotation, visualization and analysis in functional genomics research. Bioinformatics.

[31] Cuming AC, Cho SH, Kamisugi Y, Graham H, Quatrano RS (2007). Microarray analysis of transcriptional responses to abscisic acid and osmotic, salt, and drought stress in the moss, physcomitrella patens. New Phytol.

[32] Nakashima K, Ito Y, Yamaguchi-Shinozaki K (2009). Transcriptional regulatory networks in response to abiotic stresses in Arabidopsis and grasses. Plant Physiol.

[33] Zeller G, Henz SR, Widmer CK, Sachsenberg T, Ratsch G, Weigel D, Laubinger S (2009). Stress-induced changes in the Arabidopsis thaliana transcriptome analyzed using whole-genome tiling arrays. Plant J.

[34] Ma Y, Szostkiewicz I, Korte A, Moes D, Yang Y, Christmann A, Grill E (2009). Regulators of PP2C phosphatase activity function as abscisic acid sensors. Science.

[35] Xu ZY, Kim DH, Hwang I (2013). ABA homeostasis and signaling involving multiple subcellular compartments and multiple receptors. Plant Cell Rep.

[36] Park SY, Fung P, Nishimura N, Jensen DR, Fujii H, Zhao Y, Lumba S, Santiago J, Rodrigues A, Chow TFF (2009). Abscisic acid inhibits type 2C protein phosphatases via the PYR/PYL family of START proteins. Science.

[37] Fujii H, Chinnusamy V, Rodrigues A, Rubio S, Antoni R, Park SY, Cutler SR, Sheen J, Rodriguez PL, Zhu JK (2009). In vitro reconstitution of an abscisic acid signalling pathway. Nature.

[38] Kobayashi Y, Yamamoto S, Minami H, Kagaya Y, Hattori T (2004). Differential activation of the rice sucrose nonfermenting1-related protein kinase2 family by hyperosmotic stress and abscisic acid. Plant Cell.

[39] Ramirez SR, Basu C (2009). Comparative analyses of plant transcription factor databases. Curr Genomics.

[40] Kepka M, Benson CL, Gonugunta VK, Nelson KM, Christmann A, Grill E, Abrams SR (2011). Action of natural abscisic acid precursors and catabolites on abscisic acid receptor complexes. Plant Physiol.

[41] Lata C, Prasad M (2011). Role of DREBs in regulation of abiotic stress responses in plants. J Exp Bot.

[42] Wang W, Vinocur B, Shoseyov O, Altman A (2004). Role of plant heat-shock proteins and molecular chaperones in the abiotic stress response. Trends Plant Sci.

[43] Richter K, Buchner J (2001). Hsp90: Chaperoning signal transduction. J Cell Physiol.

[44] Young JC, Moarefi I, Hartl FU (2001). Hsp90: a specialized but essential protein-folding tool. J Cell Biol.

[45] Xu ZS, Li ZY, Chen Y, Chen M, Li LC, Ma YZ (2012). Heat shock protein 90 in plants: molecular mechanisms and roles in stress responses. Intern J Mol Sci.

[46] Timperio AM, Egidi MG, Zolla L (2008). Proteomics applied on plant abiotic stresses: role of heat shock proteins (HSP). J Proteomics.

[47] Haslbeck M, Franzmann T, Weinfurtner D, Buchner J (2005). Some like it hot: the structure and function of small heat-shock proteins. Nat Struct Mol Biol.

[48] Waters ER (2013). The evolution, function, structure, and expression of the plant sHSPs. J Exp Bot.

[49] Campbell JL, Klueva NY, Zheng HG, Nieto-Sotelo J, Ho THD, Nguyen HT (2001). Cloning of new members of heat shock protein HSP101 gene family in wheat (Triticum aestivum (L.) Moench) inducible by heat, dehydration, and ABA. Bba-Gene Struct Expr.

[50] Zou J, Liu A, Chen X, Zhou X, Gao G, Wang W, Zhang X (2009). Expression analysis of nine rice heat shock protein genes under abiotic stresses and ABA treatment. J Plant Physiol.

[51] Wang Y, Li J, Wang J, Li Z (2010). Exogenous H_2_O_2_ improves the chilling tolerance of manilagrass and mascarenegrass by activating the antioxidative system. Plant Growth Regul.

[52] Van Breusegem F, Vranova E, Dat JF, Inze D (2001). The role of active oxygen species in plant signal transduction. Plant Sci.

[53] Dang ZH, Zheng LL, Wang J, Gao Z, Wu SB, Qi Z, Wang YC (2013). Transcriptomic profiling of the salt-stress response in the wild recretohalophyte Reaumuria trigyna. BMC Genomics.

[54] Mittler R, Vanderauwera S, Gollery M, Van Breusegem F (2004). Reactive oxygen gene network of plants. Trends Plant Sci.

[55] Dietz KJ (2011). Peroxiredoxins in plants and cyanobacteria. Antioxid Redox Signal.

[56] Kuzniak E, Sklodowska M (2001). Ascorbate, glutathione and related enzymes in chloroplasts of tomato leaves infected by Botrytis cinerea. Plant Sci.

[57] Szalai G, Kellős T, Galiba G, Kocsy G (2009). Glutathione as an antioxidant and regulatory molecule in plants under abiotic stress conditions. J Plant Growth Regul.

[58] Kuzniak E, Sklodowska M (2004). Differential implication of glutathione, glutathione-metabolizing enzymes and ascorbate in tomato resistance to Pseudomonas syringae. J Phytopathol.

[59] Tronchet M, Balague C, Kroj T, Jouanin L, Roby D (2010). Cinnamyl alcohol dehydrogenases-C and D, key enzymes in lignin biosynthesis, play an essential role in disease resistance in Arabidopsis. Mol Plant Pathol.

[60] Mader M, Ambergfisher V (1982). Role of peroxidase in lignification of tobacco cells .1. oxidation of nicotinamide adenine-dinucleotide and formation of hydrogen-peroxide by cell-wall peroxidases. Plant Physiol.

[61] Li L, Steffens JC (2002). Overexpression of polyphenol oxidase in transgenic tomato plants results in enhanced bacterial disease resistance. Planta.

[62] Pina A, Errea P (2008). Differential induction of phenylalanine ammonia-lyase gene expression in response to in vitro callus unions of Prunus spp. J Plant Physiol.

[63] Trotel-Aziz P, Couderchet M, Vernet G, Aziz A (2006). Chitosan stimulates defense reactions in grapevine leaves and inhibits development of Botrytis cinerea. Eur J Plant Pathol.

[64] Glazebrook J (2005). Contrasting mechanisms of defense against biotrophic and necrotrophic pathogens. Annu Rev Phytopathol.

[65] Durrant WE, Dong X (2004). Systemic acquired resistance. Annu Rev Phytopathol.

[66] Hammerschmidt R, VanLoon LC (2009). Systemic Acquired Resistance. Plant Innate Immunity.

[67] Vlot AC, Dempsey DA, Klessig DF (2009). Salicylic Acid, a Multifaceted Hormone to Combat Disease. Annu Rev Phytopathol.

[68] Zimmerli L, Stein M, Lipka V, Schulze-Lefert P, Somerville S (2004). Host and non-host pathogens elicit different jasmonate/ethylene responses in Arabidopsis. Plant J.

[69] Robert-Seilaniantz A, Navarro L, Bari R, Jones JD (2007). Pathological hormone imbalances. Curr Opin Plant Biol.

[70] Yasuda M, Ishikawa A, Jikumaru Y, Seki M, Umezawa T, Asami T, Maruyama-Nakashita A, Kudo T, Shinozaki K, Yoshida S (2008). Antagonistic interaction between systemic acquired resistance and the abscisic acid-mediated abiotic stress response in Arabidopsis. Plant Cell.

[71] Jiang CJ, Shimono M, Sugano S, Kojima M, Yazawa K, Yoshida R, Inoue H, Hayashi N, Sakakibara H, Takatsuji H (2010). Abscisic acid interacts antagonistically with salicylic acid signaling pathway in rice-magnaporthe grisea interaction. Mol Plant Microbe In.

[72] Cao FY, Yoshioka K, Desveaux D (2011). The roles of ABA in plant-pathogen interactions. J Plant Res.

[73] Trapnell C, Pachter L, Salzberg SL (2009). TopHat: discovering splice junctions with RNA-Seq. Bioinformatics.

[74] Marcais G, Kingsford C (2011). A fast, lock-free approach for efficient parallel counting of occurrences of k-mers. Bioinformatics.

[75] Szklarczyk D, Franceschini A, Kuhn M, Simonovic M, Roth A, Minguez P, Doerks T, Stark M, Muller J, Bork P (2011). The STRING database in 2011: functional interaction networks of proteins, globally integrated and scored. Nucleic Acids Res.

[76] Kanehisa M, Goto S (2000). KEGG: kyoto encyclopedia of genes and genomes. Nucleic Acids Res.

[77] Robinson MD, McCarthy DJ, Smyth GK (2010). edgeR: a Bioconductor package for differential expression analysis of digital gene expression data. Bioinformatics.

